# Polymorphisms in the glucagon-like peptide-1 receptor gene and their interactions on the risk of osteoporosis in postmenopausal Chinese women

**DOI:** 10.1371/journal.pone.0295451

**Published:** 2023-12-14

**Authors:** Chang Liu, Xiaoxue Bao, Yawei Tian, Peng Xue, Yan Wang, Yukun Li

**Affiliations:** 1 Department of Endocrinology, Hebei Medical University Third Hospital, Shijiazhuang, China; 2 Key Orthopaedic Biomechanics Laboratory of Hebei Province, Orthopedic Research Institution of Hebei Province, Shijiazhuang, China; Université Clermont Auvergne - Faculté de Biologie, FRANCE

## Abstract

Postmenopausal osteoporosis (PMOP) is a prevalent form of primary osteoporosis, affecting over 40% of postmenopausal women. Previous studies have suggested a potential association between single nucleotide polymorphisms (SNPs) in glucagon-like peptide-1 receptor (GLP-1R) and PMOP in postmenopausal Chinese women. However, available evidence remains inconclusive. Therefore, this study aimed to investigate the possible association between GLP-1R SNPs and PMOP in Han Chinese women. Thus, we conducted a case-control study with 152 postmenopausal Han Chinese women aged 45–80 years, including 76 women with osteoporosis and 76 without osteoporosis. Seven SNPs of the GLP-1R were obtained from the National Center of Biotechnology Information and Genome Variation Server. We employed three genetic models to assess the association between GLP-1R genetic variants and osteoporosis in postmenopausal women, while also investigating SNP-SNP and SNP-environment interactions with the risk of PMOP. In this study, we selected seven GLP-1R SNPs (rs1042044, rs2268641, rs10305492, rs6923761, rs1126476, rs2268657, and rs2295006). Of these, the minor allele A of rs1042044 was significantly associated with an increased risk of PMOP. Genetic model analysis revealed that individuals carrying the A allele of rs1042044 had a higher risk of developing osteoporosis in the dominant model (*P* = 0.029, OR = 2.76, 95%CI: 1.09–6.99). Furthermore, a multiplicative interaction was found between rs1042044 and rs2268641 that was associated with osteoporosis in postmenopausal women (*P*_*interaction*_ = 0.034). Importantly, this association remained independent of age, menopausal duration, family history of osteoporosis, and body mass index. However, no significant relationship was observed between GLP-1R haplotypes and PMOP. In conclusion, this study suggests a close association between the A allele on the GLP-1R rs1042044 and an increased risk of PMOP. Furthermore, this risk was significantly augmented by an SNP-SNP interaction with rs2268641. These results provide new scientific insights into the development of personalized prevention strategies and treatment approaches for PMOP.

## Introduction

Osteoporosis (OP) is a prevalent metabolic bone disorder characterized by decreased bone mass, deteriorated bone microarchitecture and compromised bone strength, resulting in an increased risk of nonstress fractures [1]. It has emerged as a significant clinical and public health concern, exerting adverse effects on individuals’ health and social development. Postmenopausal OP (PMOP), which affects more than 40% of postmenopausal women [2, 3], is one of the most common forms of primary OP. Although PMOP primarily arises from estrogen deficiency following menopause, various genetic and environmental factors contribute to the development of OP [4].

Numerous studies have consistently demonstrated a strong genetic predisposition to OP [4, 5]. Warrington et al. revealed that hereditary factors could account for up to 90% of the variance in bone mineral density (BMD) [4], whereas approximately 70% of the heritability of bone turnover markers has been attributed to genetic factors [5]. Since Morrison et al.’s groundbreaking work in 1994, which identified the association of genetic polymorphisms in the vitamin D receptor with susceptibility to OP [6], a genome-wide association study has discovered multiple single nucleotide polymorphisms (SNPs) located at various genetic locus linked to the risk of OP [7]. Furthermore, evidence supports a genetic association between glucagon-like peptide-1 receptor (GLP-1R) gene polymorphisms and susceptibility to OP [8].

GLP-1R, a member of the class B1 family of G protein-coupled receptors, plays a crucial role in hormone signal reception and receptor activation through its 7-transmembrane protein structure and N-terminal extracellular domain [9]. GLP-1R exerts diverse physiological effects in various tissues, including bone. Previous studies have reported that GLP-1R-knockout mice exhibit decreased cortical bone density, thereby increasing the risk of OP [10]. The GLP-1R gene is located on chromosome 6 (6p21.2), and its biological impact is mediated by glucagon-like peptide 1 [11]. A recent study suggested that a SNP in the GLP-1R gene (rs1042044) may indirectly influence BMD [12]; specifically, the A allele of GLP-1R rs2295006 and haplotype CGAGCCA were found to be negatively correlated with BMD in the postmenopausal population [8]. However, the specific genetic polymorphisms responsible for susceptibility to PMOP remain largely unknown.

PMOP development is determined by the complex interaction between multiple genetic variants and environmental factors. However, few studies have investigated the interaction effects of these factors on the PMOP. Therefore, this study aimed to explore the associations of the GLP-1R SNPs (rs1042044, rs2268641, rs10305492, rs6923761, rs1126476, rs2268657, and rs2295006); SNP-SNP interactions; and SNP-environment interactions with the risk of PMOP among postmenopausal Han Chinese women. This study aimed to provide valuable insights into the pathogenesis and genetic diagnosis of PMOP while offering a theoretical foundation for its prevention and treatment.

## Materials and methods

### Study population

In this case-control study, we enrolled 76 women with PMOP as the case group (OP group) and 76 non-osteoporotic postmenopausal women as the control group (non-OP group), aged 45–80 years. The participants were recruited from the Endocrine Department of HEBEI MEDICAL UNIVERSITY THIRD HOSPITAL between March 23, 2023 and June 30, 2023. Before participation, all participants provided written informed consent for their involvement in this study, which was approved by the Medical Ethics Committee of HEBEI MEDICAL UNIVERSITY THIRD HOSPITAL (No.2023-026-1). We did not have access to any information that could identify individual participants during or after data collection.

### Inclusion and exclusion criteria

The inclusion criteria for the participants were as follows: a) all participants were aged 45–80 years with natural menopause having occurred more than one year earlier; b) OP diagnosis in postmenopausal women was based on the T score according to the World Health Organization criteria. T score ≥ -1standard deviation (SD) and ≤ -2.5SD was considered normal bone mineral density and OP, respectively; and c) none of the participants had received any anti-OP treatment. Participants with severe heart, liver, or kidney disease, diabetes mellitus, thyroid and parathyroid dysfunction, autoimmune disease, metabolic or hereditary bone diseases, and those using anti-OP drugs, hormones, or immunosuppressants were excluded.

### Data collection

We collected clinical and biochemical data from all participants, including age, menopausal age, height, weight, smoking or drinking habits, family history of OP, and serum levels of parathyroid hormone (PTH), 25-Hydroxyvitamin D, calcium, and phosphorus. The body mass index (BMI) was calculated using the following equation: BMI = weight/height^2^ (kg/m^2^).

### BMD measurement

The BMDs at the lumbar spine (LS), femoral neck (FN), and total hip (TH) of all participants were measured using a dual-energy X-ray absorptiometry (DXA) device (Hologic, MA, USA). In order to ensure reliability, the measurements were performed by a single technician. The coefficients of variation for LS, FN and TH were 1.14%, 1.80%, and 1.29% respectively.

### SNP selection

We identified seven GLP-1R SNPs (rs1042044, rs2268641, rs10305492, rs6923761, rs1126476, rs2268657, and rs2295006) from the National Center of Biotechnology Information (NCBI) (http://www.ncbi.nlm.nih.gov/SNP/) and Genome Variation Server (GVS) (https://gvs.gs.washington.edu/GVS150/index.jsp). The selection criteria for these tagging SNPs were as follows: a) minor allele frequency (MAF) ≥ 0.01; and b) linkage disequilibrium (LD), measured by an r^2^ value ≥ 0.33.

### DNA extraction and genotyping

Genomic DNA was extracted from peripheral blood leukocytes according to the manufacturer’s instructions provided in the genomic DNA kit (Tiangen, Beijing, China) and its concentration and quality were quantified using NanoDrop 2000 (Thermo Fisher Scientific, Waltham, MA, USA); the DNA was stored at -80°C.

The GLP-1R SNPs were genotyped using the polymerase chain reaction-ligase detection reaction (PCR-LDR). The cycling parameters consisted of an initial denaturation step at 95°C for 5 min, followed by 35 cycles of PCR including denaturation at 94°C for 20 s, annealing at 55°C for 20 s, extension at 72°C for 40 s, and final extension step at 72°C for another 40 s. Subsequently, the obtained results were processed using the 3730 XL Gene Sequencer (Applied Biosystems, MA, USA) and analyzed with the GeneMarker software version V2.6.3. The primers used to detect the GLP-1R SNPs are provided in S1 Table.

### Statistical analysis

The clinical data were analyzed using SPSS 22.0 software. The normality test of all data was verified using the Kolmogorov-Smirnov test. Continuous variables are presented as mean ± SD and were compared between groups using the Student’s t-test, while non-continuous variables are presented as median (interquartile range) and were compared using the Mann-Whitney U test. Qualitative variables are expressed as numbers (percentages) and compared using the chi-squared test. Genotype and allele frequencies, along with Hardy-Weinberg equilibrium (HWE), were assessed using the chi-squared test (*P* ≥ 0.05). LD and haplotype analysis were constructed using the SHEsis software (http://analysis.bio-x.cn/myAnalysis.php). Binary logistic regression was performed in different genetic models to analyze the associations between SNPs and PMOP. The SNP-SNP and SNP-environment interactions were assessed using the SNPStats software (https://www.snpstats.net/start.htm). Akaike Information Criterion (AIC) and Bayesian Information Criterion (BIC) were employed for model selection. The adjusted confounding factors included age, menopausal duration, family history of OP and BMI. A significance level of *P* < 0.05 was considered statistically significant for all tests.

## Results

### Clinical characteristics of all participants

Table 1 presents the demographic and clinical laboratory data of the participants. The results show significant differences in age, menopausal duration, and BMI between the OP and non-OP groups (*P* < 0.05). Moreover, patients with PMOP exhibited a higher prevalence of familial OP than the control group. There were no significant differences between groups in the menopausal age, smoking and drinking habits, and serum levels of PTH, 25-Hydroxyvitamin D, calcium and phosphorus (*P* > 0.05).

**Table 1:** Demographic and clinical characteristics of the study participants (N = 152).

Variables	Non-OP Group (n = 76)	OP Group (n = 76)	*P* Value
**Age (Years)**			
<**50**	6(7.89)	0(0.00)	0.000
**50~**	36(47.37)	18(23.68)	
**60~**	24(31.58)	29(38.16)	
**≧70**	10(13.16)	29(38.16)	
**Menopausal age (Years)**			
<**50**	27(35.53)	36(47.37)	0.138
**≧50**	49(64.47)	40(52.63)	
**Menopausal duration (Years)**			
<**10**	42(55.26)	14(18.42)	0.000
**10~**	22(28.95)	33(43.42)	
**≧20**	12(15.79)	29(38.16)	
**BMI (kg/m** ^**2**^ **)**			
**<24**	18(23.68)	36(47.37)	0.001
**24~**	32(42.11)	30(39.47)	
**≥28**	26(34.21)	10(13.16)	
**Smoking habits (Yes/No)**	2(2.63)/74(97.37)	4(5.26)/72(94.74)	0.681
**Drinking habits (Yes/No)**	12(15.79)/64(84.21)	9(11.84)/67(88.16)	0.481
**Family history of OP (Yes/No)**	15(19.74)/61(80.26)	27(35.53)/49(64.47)	0.030
**PTH (pg/ml)**	52.83(42.46–66.24)	49.96(37.67–63.59)	0.411
**25-Hydroxyvitamin D (ng/ml)**	21.28(17.07–25.48)	18.19(15.82–23.75)	0.097
**Calcium (mmol/L)**	2.27±0.11	2.26±0.13	0.642
**Phosphorus (mmol/L)**	1.22±0.17	1.22±0.18	0.887

Values are expressed as mean ± SD, median (interquartile range) or as numbers (percentages).

BMI, body mass index; OP, osteoporosis; PTH, parathyroid hormone.

### Genotypic and allelic distributions of the GLP-1R SNPs

The characteristics of the GLP-1R SNPs are presented in Table 2. For all GLP-1R SNP locus, the MAF and the HWE were used to determine whether the non-OP and OP study participants were a random sample of the target population. Thus, the selection criteria for each SNP adhered to the MAF of ≥ 0.01 and the HWE with the *P* value ≥ 0.05. In this study, GLP-1R rs10305492 was excluded because the MAF in the non-OP group was ≤ 0.01, and GLP-1R rs1126476 was excluded as it did not conform to the HWE in the non-OP group, suggesting that the two GLP-1R SNPs were underrepresented in the target population. Ultimately, this study identified five SNPs that conformed to the selection criteria. The genotypic and allelic frequencies of the GLP-1R SNPs in the study population are provided in Table 3. The genotype frequency of rs1042044 A-carrier (AC and AA) was significantly higher in the OP group than in the non-OP group (*P* = 0.017). Furthermore, the minor allele A of rs1042044 showed a significant association with an increased risk of OP (*P* = 0.016, OR = 1.75, 95%CI: 1.11–2.75). Similarly, the genotype frequencies of rs2268641 and rs2268657 T-carrier (CT and TT) were higher in the OP group than in the non-OP group (*P* < 0.05). Meanwhile, significant differences between the OP and non-OP groups were observed in the allele frequencies of rs2268641 and rs2268657 (*P* = 0.011, *P* = 0.021, respectively). Conversely, no significant differences were found in the genotypic and allelic frequencies of rs6923761 and rs2295006.

**Table 2:** The characteristics of seven GLP-1R SNPs.

SNP ID	Chr.position	Alleles	MAF in dbSNP	MAF		HWE (*P* value)		Function-Location
				Non-OP Group	OP Group	Non-OP Group	OP Group	
**rs1042044**	39073726	A/C	0.50	0.41	0.45	0.98	0.05	Nonsynon-exon7
**rs2268641**	39082490	C/T	0.36	0.36	0.49	0.98	0.49	Intron12-variant
**rs10305492**	39079018	G/A	0.01	0.00	0.01	1.00	0.95	Missense
**rs6923761**	39066296	G/A	0.01	0.02	0.02	0.86	0.86	Nonsynon-exon5
**rs1126476**	39080715	A/C	0.48	0.36	0.47	0.02	0.10	Synon-exon12
**rs2268657**	39052766	C/T	0.43	0.28	0.40	0.11	0.91	Intron1-variant
**rs2295006**	39056449	G/A	0.11	0.05	0.09	0.67	0.42	Nonsynon-exon2

SNPs, single-nucleotide polymorphism; MAF, minor allele frequency. dbSNP, the Genetic Variation Database of the National Center for Biotechnology Information; HWE, Hardy-Weinberg equilibrium; OP, osteoporosis.

**Table 3:** Genotypic and allelic frequencies of the GLP-1R SNPs.

SNPs	Genotypes/Alleles	Non-OP Group (n = 76)	OP Group (n = 76)	χ2	*P* Value	OR(95%CI)
**rs1042044**	CC	26(34.2)	11(14.5)	8.182	0.017	-
	AC	37(48.7)	46(60.5)			
	AA	13(17.1)	19(25.0)			
	C	89(58.6)	68(44.7)	5.809	0.016	1.75(1.11–2.75)
	A	63(41.4)	84(55.3)			
**rs2268641**	CC	31(40.8)	17(22.4)	6.843	0.033	-
	CT	35(46.0)	41(53.9)			
	TT	10(13.2)	18(23.7)			
	C	97(63.8)	75(49.3)	6.481	0.011	1.81(1.14–2.87)
	T	55(36.2)	77(50.7)			
**rs6923761**	GG	73(96.1)	73(96.1)	0.000	1.000	-
	AG	3(3.9)	3(3.9)			
	AA	0(0.0)	0(0.0)			
	G	149(98.0)	149(98.0)	0.000	1.000	1.00(0.20–5.04)
	A	3(2.0)	3(2.0)			
**rs2268657**	CC	37(48.7)	27(35.5)	6.976	0.031	-
	CT	36(47.4)	37(48.7)			
	TT	3(3.9)	12(15.8)			
	C	110(72.4)	91(59.9)	5.301	0.021	1.76(1.09–2.84)
	T	42(27.6)	61(40.1)			
**rs2295006**	GG	69(90.8)	63(82.9)	2.073	0.150	-
	AG	7(9.2)	13(17.1)			
	AA	0(0.0)	0(0.0)			
	G	145(95.4)	139(91.4)	1.927	0.165	1.94(0.75–5.00)
	A	7(4.6)	13(8.6)			

SNPs, single-nucleotide polymorphism; OP, osteoporosis; OR, odds ratio; CI, confidence interval.

### Effects of genotype on the risk of PMOP

The effect of the GLP-1R SNPs on the risk of PMOP was further investigated using binary logistic regression analysis, after adjusting for confounding factors including age, menopausal duration, family history of OP, and BMI (Table 4). In the dominant model, individuals with the AC-AA genotype of rs1042044 exhibited a significantly higher risk of PMOP than those with the homozygous CC genotype (adjusted *P* = 0.029, OR = 2.76, 95% CI: 1.09–6.99). However, owing to the limited sample size, no association was observed between rs2268641 and PMOP risk. Moreover, in the recessive model, individuals with the homozygous TT genotype of rs2268657 showed an increased risk of PMOP (*P* = 0.012), although this difference was insignificant after adjusting for confounders (*P* = 0.053). No significant relationship between the GLP-1R SNPs and PMOP risk was found in the other genetic models examined in this study. Based on the AIC and BIC evaluations, the dominant model emerged as the optimal choice among the three GLP-1R SNP models.

**Table 4:** Genetic model analysis of the association between GLP-1R SNPs and PMOP risk.

Models	Genotypes	Non-OP Group	OP Group	OR(95%CI)	*P* value	AIC	BIC	OR(95%CI)*	P*value	AIC*	BIC*
**rs1042044**											
**Dominant**	CC	26(34.2)	11(14.5)	1	0.004	206.5	212.5	1	0.029	177.5	207.8
	AC-AA	50(65.8)	65(85.5)	3.07(1.39–6.81)				2.76(1.09–6.99)			
**Recessive**	CC-AC	63(82.9)	57(75.0)	1	0.23	213.3	219.3	1	0.53	181.9	212.2
	AA	13(17.1)	19(25.0)	1.62(0.73–3.56)				1.35(0.53–3.45)			
**Overdominant**	CC-AA	39(51.3)	30(39.5)	1	0.14	212.6	218.6	1	0.17	180.4	210.7
	AC	37(48.7)	46(60.5)	1.62(0.85–3.08)				1.73(0.79–3.79)			
**rs2268641**											
**Dominant**	CC	31(40.8)	17(22.4)	1	0.014	208.7	214.7	1	0.23	178.9	208.1
	CT-TT	45(59.2)	59(77.6)	2.39(1.18–4.85)				1.67(0.72–3.91)			
**Recessive**	CC-CT	66(86.8)	58(76.3)	1	0.092	211.9	217.9	1	0.10	179.7	209.9
	TT	10(13.2)	18(23.7)	2.05(0.88–4.79)				2.22(0.83–5.93)			
**Overdominant**	CC-TT	41(53.9)	35(46.1)	1	0.33	213.8	219.8	1	0.81	182.3	212.5
	CT	35(46.1)	41(53.9)	1.37(0.73–2.60)				0.91(0.43–1.95)			
**rs2268657**											
**Dominant**	CC	37(48.7)	27(35.5)	1	0.10	207.2	212.1	1	0.06	177.8	208.0
	CT-TT	39(51.3)	49(64.5)	1.72(0.90–3.30)				2.12(0.96–4.66)			
**Recessive**	CC-CT	73(96.0)	64(84.2)	1	0.012	208.3	214.4	1	0.053	178.6	208.8
	TT	3(4.0)	12(15.8)	4.56(1.23–16.89)				3.82 (0.89–16.37)			
**Overdominant**	CC-TT	40(52.6)	39(51.3)	1	0.87	214.7	220.7	1	0.48	181.8	212.1
	CT	36(47.4)	37(48.7)	1.05(0.56–1.99)				1.32 (0.61–2.88)			

OP, osteoporosis; OR, odds ratio; CI, confidence interval; AIC, Akaike Information Criterion; BIC, Bayesian Information Criterion.

* Adjusted the age, menopausal duration, family history of OP and BMI.

### Association of GLP-1R SNP-SNP and SNP-environment multiplicative interactions with PMOP risk

To investigate the GLP-1R SNP-SNP multiplicative interactions in both the OP and non-OP groups, we employed the dominant model as the optimal genetic model. Our findings revealed a significant association between the multiplicative interaction of rs1042044 and rs2268641 and PMOP risk (adjusted *P*_*interaction*_ = 0.034), as shown in Table 5. Additionally, we thoroughly examined the multiplicative interactions between the GLP-1R SNPs and clinically relevant characteristics of OP in postmenopausal women. However, no statistically significant associations were observed between these interactions and the PMOP risk (*P*_*interaction*_ > 0.05) (Table 6).

**Table 5:** Association between GLP-1R SNP-SNP multiplicative interaction and PMOP risk.

SNP*SNP		Non-OP Group	OP Group	OR(95%CI)	*P*_*interaction*_
rs1042044*rs2268641					
**CC**	**CC**	12	7	1.00	0.034
**AC-AA**	**CC**	19	10	0.79(0.19–3.34)	
**CC**	**CT-TT**	14	4	0.28(0.05–1.60)	
**AC-AA**	**CT-TT**	31	55	1.91(0.58–6.35)	
rs1042044*rs2268657					
**CC**	**CC**	12	5	1.00	0.73
**AC-AA**	**CC**	25	22	2.19(0.56–8.54)	
**CC**	**CT-TT**	14	6	1.56(0.30–8.17)	
**AC-AA**	**CT-TT**	25	43	4.78(1.27–17.99)	
rs2268641*rs2268657					
**CC**	**CC**	14	9	1.00	0.10
**CT-TT**	**CC**	23	18	0.74(0.21–2.59)	
**CC**	**CT-TT**	17	8	0.74(0.17–3.24)	
**CT-TT**	**CT-TT**	22	41	2.33(0.71–7.70)	

SNPs, single-nucleotide polymorphism; OP, osteoporosis; OR, odds ratio; CI, confidence interval.

*P*_*interaction*,_ adjusted the age, menopausal duration, family history of OP and BMI.

**Table 6:** Association between SNP-environment multiplicative interaction and PMOP risk (*P* value).

	Age	Menopausal duration	Family history of OP	BMI
**rs1042044**				
**P-dominant**	0.97	0.14	0.10	0.32
**P-recessive**	0.77	0.41	0.54	0.50
**P-overdominant**	0.72	0.54	0.076	0.19
**rs2268641**				
**P-dominant**	0.92	0.31	0.062	0.056
**P-recessive**	0.61	0.71	0.053	0.29
**P-overdominant**	0.53	0.20	0.11	0.15
**rs2268657**				
**P-dominant**	0.90	0.72	0.34	0.65
**P-recessive**	0.62	0.54	0.28	0.46
**P-overdominant**	1.00	0.98	0.26	0.21

OP, osteoporosis; BMI, body mass index; *P*, adjusted confounding factors.

### Association between GLP-1R SNP haplotypes and PMOP risk

**Fig 1:**
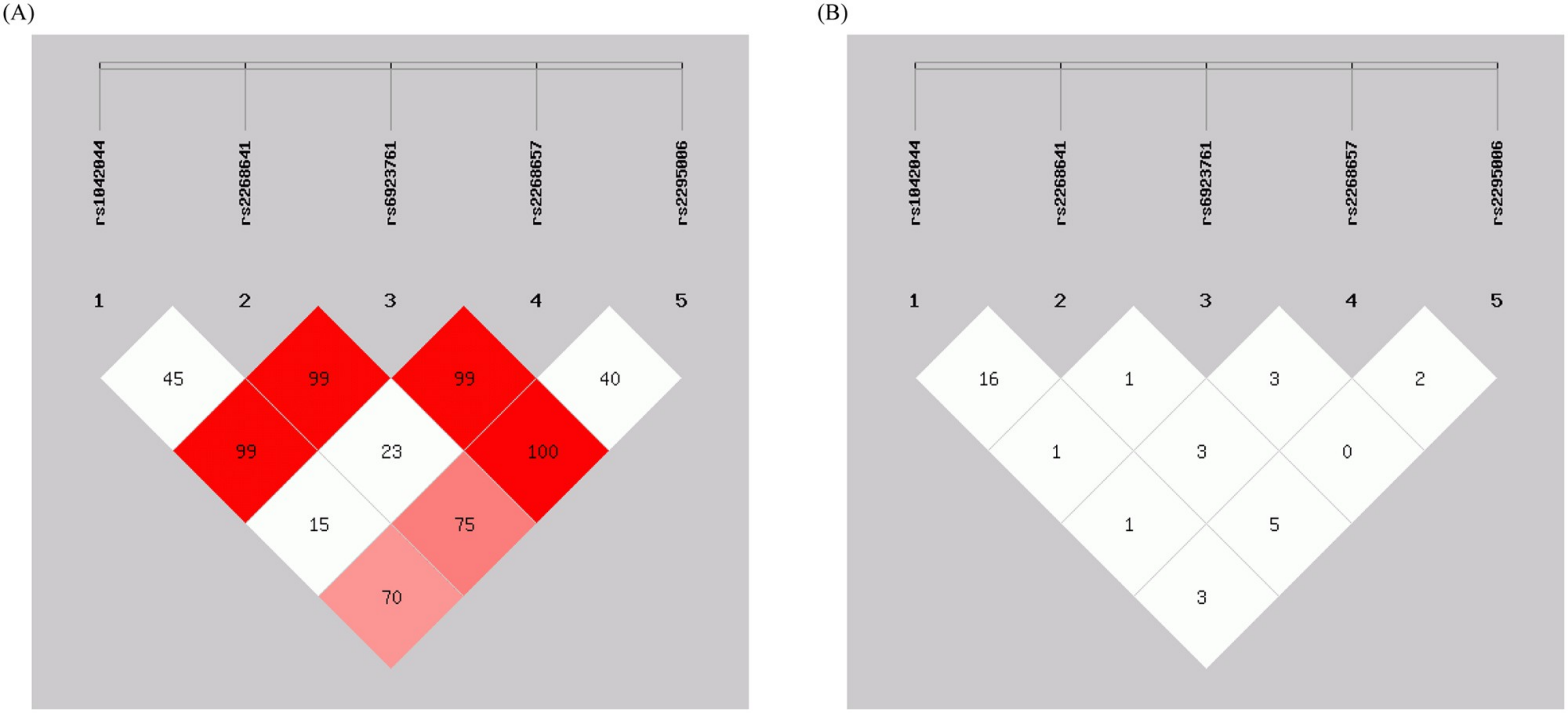
Linkage disequilibrium diagram among five GLP-1R SNPs. (A) Linkage disequilibrium D’ value between SNPs. The higher the value, the darker the color of the block, the stronger the correlation of the linkage disequilibrium. (B) Linkage disequilibrium r^2^ value between SNPs. When both D’ and r^2^ values are 0, it signifies linkage equilibrium. For example, let’s examine whether rs1042044 and rs2295006 are in linkage disequilibrium. We trace the column from rs1042044 to rs2295006 and vice versa. As we can observe, there is a D’ value of 0.7 and an r^2^ value of 0.03, suggesting that rs1042044 and rs2295006 are in higher linkage disequilibrium with each other.

SHEsis software was utilized for the analysis of the LD, as depicted in Fig 1. The findings indicate that the five GLP-1R SNPs are in LD with each other, enabling the construction of haplotypes within this linkage domain block. In this haplotype analysis, the CCGCG haplotype displayed the highest frequency and was selected as the reference group. However, even after adjusting for confounding factors, no significant association was observed between haplotypes and PMOP risk (*P* > 0.05) (Table 7).

**Table 7:** Association between GLP-1R SNP haplotypes and PMOP risk (Frequence>1%).

	rs1042044	rs2268641	rs6923761	rs2268657	rs2295006	Frequence	*P* value	OR(95%CI)
**1**	C	C	G	C	G	0.268	-	1.00
**2**	A	T	G	C	G	0.147	0.53	0.69(0.22–2.18)
**3**	A	C	G	C	G	0.131	0.37	0.52(0.12–2.16)
**4**	A	T	G	T	G	0.104	0.077	3.45(0.88–13.48)
**5**	C	C	G	T	G	0.089	0.31	0.36(0.05–2.62)
**6**	C	T	G	C	G	0.087	0.26	0.45(0.11–1.83)
**7**	A	C	G	T	G	0.047	0.36	2.81(0.31–5.38)
**8**	C	T	G	T	G	0.042	0.23	1.26(0.40–4.85)
**9**	A	T	G	T	A	0.037	0.67	1.65(0.16–6.87)
**10**	C	C	A	T	G	0.020	0.51	2.20(0.21–5.03)

OR, odds ratio; CI, confidence interval; Adjusted the age, menopausal duration, family history of OP and BMI.

## Discussion

In recent years, there has been growing interest in understanding the role of GLP-1R gene polymorphisms in the development of OP. However, existing studies on this topic are limited in terms of the number of SNPs examined. This limitation hinders our comprehensive understanding of how genetic variations in GLP-1R contribute to OP risk. Moreover, it is important to note that different research studies have reported heterogeneous associations between these genetic variations and OP. These discrepancies could be attributed to various factors such as differences in study design, ethnicity, geographical region, and sample size. To address these gaps in knowledge, we conducted a study specifically focusing on postmenopausal women of the Han Chinese ethnicity, a population known to be at higher risk of developing OP. By examining seven specific GLP-1R polymorphisms, we aimed to provide additional insights into their potential effects on PMOP. Our results revealed an interesting finding regarding one particular SNP, rs1042044, which showed a positive effect on PMOP risk among our study participants. Specifically, we found that individuals carrying the minor allele A of rs1042044 had a significantly increased risk of developing PMOP compared to those without this allele. Furthermore, when analyzing the data using the dominant model, individuals with rs1042044 exhibited an even higher risk of PMOP than those without this variant. Notably, GLP-1R SNP rs2268641 did not demonstrate any significant association with PMOP risk when analyzed alone; however, we observed a synergistic interaction between rs1042044 and rs2268641 that further augmented the overall risk of developing PMOP.

Recently, several studies have investigated the biological effects of GLP-1R SNPs. GLP-1R rs1042044 was found to be associated with morning salivary cortisol levels in preschoolers [13], reward learning, and anhedonia [14]. A study also reported that the rs1042044 C>A polymorphism may influence the risk of papillary thyroid cancer by affecting GLP-1R expression [15]. Additionally, GLP-1R rs2268641 has been linked to obesity-related traits in European Americans [16]. However, Michałowska et al. did not observe any significant association between the variants rs2268641 and rs6923761 and metabolic syndrome in a Polish cohort [17]. GLP-1R rs6923761 was strongly correlated with fasting serum GLP-1 levels in patients with initial T2DM; specific variants were related to weight loss outcomes [18], as well as the efficacy of liraglutide, a GLP-1 receptor agonist [19]. Research has shown that certain SNPs within the GLP-1R gene are associated with specific ethnic populations. For instance, the GLP-1R rs2268657 gene variant was significantly linked to the rate of gastric emptying in Caucasian men [20].

To date, only a limited number of studies have investigated the effect of the GLP-1R SNPs on OP. The findings of a study involving 427 Chinese nuclear families with male offspring demonstrated a significant association between rs1042044 and both lean tissue and adipose tissue. Moreover, high levels of whole-body fat and lean tissue content positively correlated with peak BMDs at the LS, FN, and TH [12], suggesting that rs1042044 may indirectly influence BMD. A negative correlation was observed between the rs2295006 A/A genotype and CGAGCCA haplotype and BMDs, as reported in another study [8]. However, further research is needed to establish conclusive evidence regarding the relationship between the GLP-1R SNPs and PMOP.

In the present study, we observed an association between GLP-1R rs1042044-A and an increased risk of PMOP in a specific population (such as that in Hebei Province). However, the precise mechanism underlying this association between GLP-1R polymorphisms and PMOP risk remains elusive. We hypothesize that the increased susceptibility to OP in carriers of the rs1042044-A allele may primarily be mediated through alterations in bone metabolism. It is well known that bone remodeling is a complex process involving osteoblast-mediated bone formation and osteoclast-mediated bone resorption to maintain skeletal homeostasis. Osteoblasts are responsible for synthesizing new bone matrix, whereas osteoclasts are involved in breaking down old or damaged bone tissue. The balance between these two processes is crucial for maintaining healthy bones. Previous studies have demonstrated the expression of GLP-1R on various cell types including osteoblast-like MC3T3-E1 cells [21, 22], bone marrow stem cells [23], primary osteoblasts, and osteoclasts [24]. This suggests that GLP-1R plays a significant role in regulating the differentiation of both osteoblasts and osteoclasts. Furthermore, our previous investigations have also confirmed the presence of GLP-1R on MC3T3-E1 cells resembling osteoblasts [25–27], as well as on murine bone marrow-derived macrophage cells and RAW264.7 preosteoclasts [28]. Therefore, it can be inferred that GLP-1R plays a crucial role in regulating the differentiation of both osteoblasts and osteoclasts.

GLP-1R rs1042044, a nonsynonymous SNP located on exon 7 of the GLP-1R gene, results in the substitution of adenine with cytosine at position 260 within the intracellular loops of GLP-1R protein, consequently the replacement of leucine with phenylalanine (S1 Fig). Previous studies have demonstrated that GLP-1R rs1042044 can affect the expression and function of GLP-1R protein [13, 29]. As a member of the class B1 G protein-coupled receptor family, GLP-1R exhibits specific binding affinity for GLP-1, initiating cAMP production as the primary signal transduction pathway [9]. Importantly, downstream receptor signaling is largely determined by these intracellular loops [30]. Therefore, it is possible that GLP-1R rs1042044 may affect GLP-1R function by altering both binding affinity for GLP-1 and intracellular signaling following hormone-receptor binding [30]. Furthermore, it is noteworthy that rs1042044 might not act alone but could also be influenced by other nearby genetic variations [13, 30]. These linked variations may contribute to differences in GLP-1R function through effects on post-translational modifications or interactions with co-regulatory proteins. Based on these findings, we propose that reduced levels of GLP-1R expression caused by the rs1042044 variation could have implications for bone health. Animal studies have demonstrated that mice lacking functional GLP-1Rs exhibit weaker bones associated with decreased collagen cross-linking [31]. Considering these observations, it is plausible to suggest that individuals carrying the rs1042044-A allele might face an increased risk of developing OP owing to potential influences on both bone quality and bone turnover. However, further investigations are required to understand precisely how this genetic variant affects bone metabolism and whether there are any additional contributing factors.

The occurrence and progression of PMOP are influenced by both genetic variations and environmental factors. While hereditary factors only contribute partially to the disease, SNP-SNP and SNP-environment interactions play a crucial role in determining an individual’s susceptibility to this condition [32]. However, there has been limited research conducted on the specific interaction between GLP-1R SNP-SNP and SNP-environment regarding the risk of PMOP. Therefore, further exploration is needed to understand the underlying mechanisms fully. Our recent findings have revealed that there is indeed a statistically significant interaction effect between rs1042044 and rs2268641, which significantly increases the risk of PMOP. Interestingly, although the rs2268641 variant alone does not affect PMOP risk, its combined effect with the rs1042044 variant is significantly higher. The T allele variant at rs2268641 may increase the risk of PMOP in postmenopausal Han Chinese women who carry the A allele variant at rs1042044.

GLP-1R rs2268641 variant is located within intron 12 of the GLP-1R gene. Although a study has revealed a significant association between GLP-1R rs2268641 and obesity among European Americans [16], the available data do not provide conclusive evidence on how rs2268641 affects GLP-1R expression and function. Introns, non-coding DNA sequences, undergo splicing during gene transcription and do not participate in the process of protein synthesis. Nevertheless, introns exert a significant influence on various steps of mRNA maturation, encompassing transcription initiation, transcription elongation, transcription termination, polyadenylation, nuclear export, and mRNA stability [33]. These steps are pivotal for gene transcription and expression and may potentially induce the emergence of novel mRNA through evolutionary processes. Thus, we speculate GLP-1R rs2268641 may influence the expression of GLP-1R gene by potentially modifying the gene transcription and inducing the production of a new mRNA encoding for a non-functional protein. Furthermore, the association of rs1042044 and rs2268641 may also derive from the effects of causal variants that are in linkage disequilibrium with them. In brief, these findings emphasize how different genetic variations can interact with each other and potentially modify an individual’s susceptibility to developing PMOP. A better understanding of how rs1042044 and rs2268641 variants influence disease risk and their successful treatment would significantly impact precision medicine development, and understanding these complex interactions may pave the way for personalized prevention strategies or targeted treatments based on an individual’s unique genetic profile.

In conclusion, our findings suggested that the GLP-1R rs1042044 gene polymorphism was associated with an increased risk of PMOP. Moreover, a significant SNP-SNP interaction between rs1042044 and rs2268641 contributed to the development of PMOP. This study underscores the significance of precision medicine in tailoring treatments and prevention strategies for patients with PMOP based on their genetic background, aimed at identifying high-risk individuals and improving therapeutic success rates. Further investigation into the mechanistic role of these polymorphisms may enhance our understanding of OP etiology and development. Understanding how genetic variations such as rs1042044-A affect GLP-1R function can provide insights into potential therapeutic targets for preventing or treating PMOP. Further research is needed to elucidate the exact molecular mechanisms by which this polymorphism influences bone metabolism and contributes to increased PMOP risk.

However, this study had a few limitations. First, the sample size was insufficient to achieve adequate statistical power for this study, and the participants were from a specific region, limiting the generalizability of our findings to other clinical phenotypes affected by these variants. Therefore, larger prospective studies are needed to validate our results. Second, we only investigated seven GLP-1R SNPs in postmenopausal women, which may not represent the full spectrum of GLP-1R SNPs. Thus, further research is warranted to explore whether other GLP-1R SNPs also contribute to the risk of PMOP. Lastly, the underlying molecular mechanisms through which these genetic variants influence PMOP remain unknown and require further investigation.

## Conclusions

In conclusion, our study suggested that the A allele on the GLP-1R rs1042044 gene locus was closely associated with an increased risk of PMOP, and this risk was significantly increased with an SNP-SNP interaction with rs2268641. These results provide scientific evidence for further development of personalized prevention strategies and treatment approaches.

## Supporting information

S1 Fig The schematic diagram of human GLP-1R protein. The location of GLP-1R rs1042044 polymorphic variance is highlighted in red. F, phenylalanine.
(PDF)

S1 Table The primers used for GLP-1R SNPs. (DOCX)

S1 File (XLSX)
