# Supplementary figures and images for: Polymorphisms in the glucagon-like peptide-1 receptor gene and their interactions on the risk of osteoporosis in postmenopausal Chinese women
Source: PLoS One. 2023 Dec 14;18(12):e0295451. doi: 10.1371/journal.pone.0295451 (PMC10721101; doi:10.1371/journal.pone.0295451)

GLP-1R

NH<sub>2</sub>

Extracellular

Intracellular

F

260

rs1042044

COOH

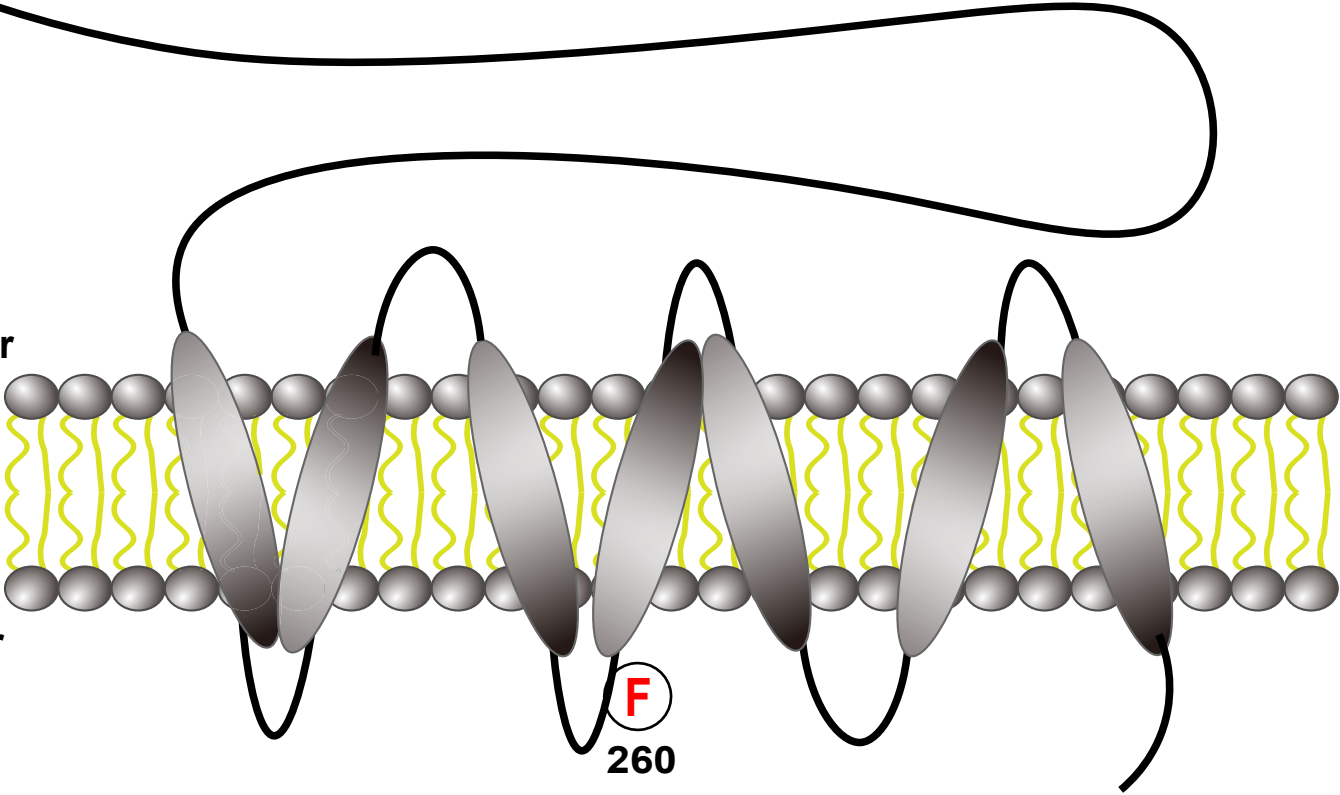

Supplement: S1 Fig — The location of GLP-1R rs1042044 polymorphic variance is highlighted in red. F, phenylalanine. (PDF) [file pone.0295451.s001.pdf]
