# Supplementary material for: Polymorphisms in the glucagon-like peptide-1 receptor gene and their interactions on the risk of osteoporosis in postmenopausal Chinese women
Source: PLoS One. 2023 Dec 14;18(12):e0295451. doi: 10.1371/journal.pone.0295451 (PMC10721101; doi:10.1371/journal.pone.0295451)
Supplement: S1 Table — (DOCX) [file pone.0295451.s002.docx]

**S1 Table. The primers used for GLP-1R SNPs.**

| **SNPs** | **Primers sequences** | **Product length (bp)** |
| --- | --- | --- |
| **rs1042044** | Forward primer： 5′-GTTTCTGCTCATGCAGTACT-3′ | 146 |
|  | Reverse primer： 5′-GGTTCTTACCCCAGCCTATG-3′ |  |
| **rs2268641** | Forward primer： 5′-CAAGATCCCACTTAATGCCTCAG-3′ | 145 |
|  | Reverse primer： 5′-CCTGCCCTACCCTCCAAAC-3′ |  |
| **rs10305492** | Forward primer： 5′-GAACTCCAACATGAACTACTG-3′ | 243 |
|  | Reverse primer： 5′-AGTTACATCACCTGCATTTG-3′ |  |
| **rs6923761** | Forward primer： 5′-CTCCTGTTCCTCTACATCATCTA-3′ | 158 |
|  | Reverse primer： 5′-GCTGCTTCATTCCTCTATCTG-3′ |  |
| **rs1126476** | Forward primer： 5′-CTCCTGCTTCCTCCCTCTT-3′ | 155 |
|  | Reverse primer： 5′-CCCCATTGTACTACTCCAGAC-3′ |  |
| **rs2268657** | Forward primer： 5′-GCTTGAGAAGTCACAAAGATTT-3′ | 145 |
|  | Reverse primer： 5′-ACTGTGGGACACGATTTATC-3′ |  |
| **rs2295006** | Forward primer： 5′-GCCTCCCATATATGCCCTC-3′ | 140 |
|  | Reverse primer： 5′-AGCCTACATGGACTCACC-3′ |  |

SNPs, single-nucleotide polymorphism.
